# *N*-[Amino­(azido)­meth­ylidene]-4-methyl­benzene­sulfonamide

**DOI:** 10.1107/S1600536811026973

**Published:** 2011-07-23

**Authors:** Ayyaz Mahmood, Islam Ullah Khan, Muhammad Nadeem Arshad, Jamil Ahmed

**Affiliations:** a Materials Chemistry Laboratory, Department of Chemistry, GC University, Lahore 54000, Pakistan

## Abstract

In the title mol­ecule, C_8_H_10_N_5_O_2_S, the amino­(azido)­methyl and *p*-toluene­sulfonyl moieties are inclined almost at right angles with respect to each other, making a dihedral angle of 83.49 (6)°. An intra­molecular N—H⋯O hydrogen bond gives rise to the formation of six-membered ring with graph-set motif *S*(6). In the crystal, inter­molecular N—H⋯O hydrogen bonding is responsible for the formation of dimers about inversion centers, which are linked through another N—H⋯O inter­action along the *b* axis.

## Related literature

For the synthesis, see: Arshad *et al.* (2009 ▶). For the biological activity of sulfonamides, see: Moree *et al.* (1991 ▶); Arshad *et al.* (2008 ▶); Gennarti *et al.* (1994 ▶). For related structures, see: Denny *et al.* (1980 ▶); Müller & Bärnighausen (1970 ▶). For graph-set notation, see: Bernstein *et al.* (1995 ▶).
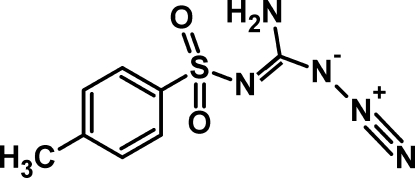


## Experimental

### 

#### Crystal data

C_8_H_9_N_5_O_2_S
*M* *_r_* = 239.26
Triclinic, 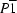

*a* = 6.8986 (2) Å
*b* = 7.2146 (2) Å
*c* = 11.3771 (3) Å
α = 92.244 (1)°
β = 93.615 (1)°
γ = 110.505 (1)°
*V* = 528.18 (3) Å^3^
*Z* = 2
Mo *K*α radiation
μ = 0.30 mm^−1^
*T* = 296 K
0.34 × 0.17 × 0.17 mm

#### Data collection

Bruker APEXII CCD diffractometer
8664 measured reflections
2549 independent reflections
2343 reflections with *I* > 2σ(*I*)
*R*_int_ = 0.020

#### Refinement

*R*[*F* ^2^ > 2σ(*F* ^2^)] = 0.031
*wR*(*F* ^2^) = 0.088
*S* = 1.09
2549 reflections
152 parameters
H atoms treated by a mixture of independent and constrained refinement
Δρ_max_ = 0.30 e Å^−3^
Δρ_min_ = −0.29 e Å^−3^

### 

Data collection: *APEX2* (Bruker, 2007 ▶); cell refinement: *SAINT* (Bruker, 2007 ▶); data reduction: *SAINT*; program(s) used to solve structure: *SHELXS97* (Sheldrick, 2008 ▶); program(s) used to refine structure: *SHELXL97* (Sheldrick, 2008 ▶); molecular graphics: *ORTEP-3 for Windows* (Farrugia, 1997 ▶); software used to prepare material for publication: *WinGX* (Farrugia, 1999 ▶).

## Supplementary Material

Crystal structure: contains datablock(s) I, global. DOI: 10.1107/S1600536811026973/pv2426sup1.cif

Structure factors: contains datablock(s) I. DOI: 10.1107/S1600536811026973/pv2426Isup2.hkl

Supplementary material file. DOI: 10.1107/S1600536811026973/pv2426Isup3.cml

Additional supplementary materials:  crystallographic information; 3D view; checkCIF report

## Figures and Tables

**Table 1:** Hydrogen-bond geometry (Å, °)

*D*—H⋯*A*	*D*—H	H⋯*A*	*D*⋯*A*	*D*—H⋯*A*
N2—H2*N*⋯O2^i^	0.80 (2)	2.24 (2)	2.9459 (16)	148 (2)
N2—H3*N*⋯O1^ii^	0.89 (2)	2.08 (2)	2.9481 (15)	164 (2)
N2—H2*N*⋯O2	0.80 (2)	2.34 (2)	2.8862 (16)	127 (2)

Symmetry codes: (i) 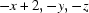; (ii) 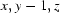.

**Fig. 1.**
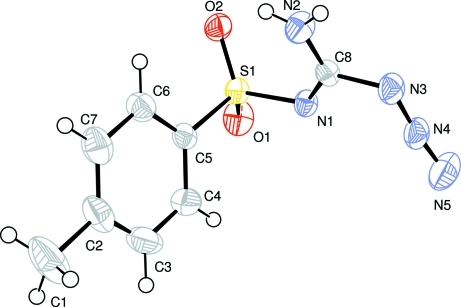
Labelled diagram of the title molecule with thermal ellipsoids drawn at 50% probability level.

**Fig. 2.**
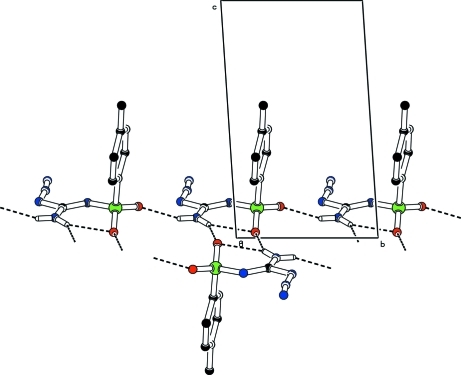
Unit cell packing diagram of the title compound showing hydrogen bonds by dshed lines; H-atoms not involved in H-bonds have been excluded for clarity.

## supplementary crystallographic information

### Comment

Sulfonamides are an important class of pharmaceutical compounds (Moree *et al.*, 1991), they exhibit a broad spectrum of biological activites which include antibacterial, diuretic, hypoglycermic, anti-convulsant, HIV protease inhibitors and for the treatment of inflammatory rheumatic and non-rheumatic processes including onsets and traumatologic lesions (Arshad *et al.*, 2008; Gennarti *et al.*, 1994). Herein, we report the crystal structure of the title compound.

In the title compound (Fig. 1), the azido group consisting of three nitrogen atoms carries cationic and anionic characters (Denny *et al.*, 1980; Muller & Barnighausen, 1970). The bond distance N4—N5 is 1.112 (2) Å, which is nearly equal to a ≡bond distance between two nitrogen atoms *i.e.* 1.10 Å. The amino(azido)methyl (N1/C8/N2/N3/N4/N5) moiety is almost planer with r. m. s. deviation of 0.0156 Å and is oriented at a dihedral angle of 83.19 (5)° with respect to the toluene ring (C1–C7). The molecule exhibits both inter- and intra-molecular hydrogen bonding. The intermolecular hydrogen bonds result in dimers about inversion centers which are further connected through N—H···O type interactions and extended along the *b* axis (Tab. 1 & Fig. 2). The intramolecular hydrogen bonding N2—H2N···O2 gives rise to the formation of a six membered ring motif which can be represented mathematically as *S*_1_^1^(6) (Bernstein *et al.*, 1995).

### Experimental

A mixture of 5-aminotetrazole monohydrate (4.85 mmol, 0.5 g) and *p*-toluenesulfonyl chloride (4.85 mmol, 0.92 g) was stirred in distilled water (10 ml) at room temperature while pH was maintained at 9–10 in accordance with (Arshad *et al.*, 2009). The completion of the reaction was checked by TLC. As the reaction completed, the precipitates obtained were filtered, washed with distilled water and finally dried. Suitable crystals for X-ray analysis were grown from mixture of methanol and ethyl acetate (1:1) by slow evaporation. Yield of the reaction was 84% (0.97 g). mp 408–413 K.

### Refinement

All H atoms were positioned geometrically with C_methyl_—H = 0.96 Å, C_aromatic_—H = 0.93 Å & N1—H = 0.8600 Å and treated as riding on their parent atoms with *U*_iso_(H) = 1.2*U*_eq_ for aromatic & N1 H-atoms and 1.5*U*_eq_ for methyl H-atoms. The hydrogen atoms for primary amino group were located *via* fourier map allowed to refine with *U*_iso_(H) = 1.5 *U*_eq_(N).

### Crystal data

**Table:**

C_8_H_9_N_5_O_2_S	*Z* = 2
*M**_r_* = 239.26	*F*(000) = 248
Triclinic, *P*1	*D*_x_ = 1.504 Mg m^−^^3^
Hall symbol: -P 1	Melting point: 444(2) K
*a* = 6.8986 (2) Å	Mo *K*α radiation, λ = 0.71073 Å
*b* = 7.2146 (2) Å	Cell parameters from 7120 reflections
*c* = 11.3771 (3) Å	θ = 3.0–28.3°
α = 92.244 (1)°	µ = 0.30 mm^−^^1^
β = 93.615 (1)°	*T* = 296 K
γ = 110.505 (1)°	Needle, colourless
*V* = 528.18 (3) Å^3^	0.34 × 0.17 × 0.17 mm

### Data collection

**Table:**

Bruker APEXII CCD diffractometer	2343 reflections with *I* > 2σ(*I*)
Radiation source: fine-focus sealed tube	*R*_int_ = 0.020
graphite	θ_max_ = 28.3°, θ_min_ = 3.2°
φ and ω scans	*h* = −9→9
8664 measured reflections	*k* = −9→9
2549 independent reflections	*l* = −15→14

### Refinement

**Table:**

Refinement on *F*^2^	Primary atom site location: structure-invariant direct methods
Least-squares matrix: full	Secondary atom site location: difference Fourier map
*R*[*F*^2^ > 2σ(*F*^2^)] = 0.031	Hydrogen site location: inferred from neighbouring sites
*wR*(*F*^2^) = 0.088	H atoms treated by a mixture of independent and constrained refinement
*S* = 1.09	*w* = 1/[σ^2^(*F*_o_^2^) + (0.0384*P*)^2^ + 0.179*P*] where *P* = (*F*_o_^2^ + 2*F*_c_^2^)/3
2549 reflections	(Δ/σ)_max_ = 0.004
152 parameters	Δρ_max_ = 0.30 e Å^−^^3^
0 restraints	Δρ_min_ = −0.29 e Å^−^^3^

### Special details

**Table:**

Geometry. All esds (except the esd in the dihedral angle between two l.s. planes) are estimated using the full covariance matrix. The cell esds are taken into account individually in the estimation of esds in distances, angles and torsion angles; correlations between esds in cell parameters are only used when they are defined by crystal symmetry. An approximate (isotropic) treatment of cell esds is used for estimating esds involving l.s. planes.
Refinement. Refinement of *F*^2^ against ALL reflections. The weighted *R*-factor *wR* and goodness of fit *S* are based on *F*^2^, conventional *R*-factors *R* are based on *F*, with *F* set to zero for negative *F*^2^. The threshold expression of *F*^2^ > 2σ(*F*^2^) is used only for calculating *R*-factors(gt) *etc*. and is not relevant to the choice of reflections for refinement. *R*-factors based on *F*^2^ are statistically about twice as large as those based on *F*, and *R*- factors based on ALL data will be even larger. The reflection 0 0 1 has been omitted as this was obscured by beamstop.

### Fractional atomic coordinates and isotropic or equivalent isotropic displacement parameters (Å^2^)

**Table:**

	*x*	*y*	*z*	*U*_iso_*/*U*_eq_	
S1	0.77484 (4)	0.16023 (4)	0.12702 (3)	0.02808 (11)	
O1	0.70020 (17)	0.32207 (14)	0.13239 (11)	0.0459 (3)	
O2	0.87648 (15)	0.13621 (15)	0.02394 (8)	0.0370 (2)	
N1	0.57940 (16)	−0.03376 (15)	0.14825 (9)	0.0291 (2)	
N2	0.7254 (2)	−0.27167 (18)	0.08497 (12)	0.0383 (3)	
H2N	0.825 (3)	−0.194 (3)	0.0593 (18)	0.057*	
H3N	0.710 (3)	−0.399 (3)	0.0848 (18)	0.057*	
N3	0.40903 (18)	−0.37488 (16)	0.15282 (11)	0.0368 (3)	
N4	0.27278 (18)	−0.32615 (17)	0.19944 (11)	0.0381 (3)	
N5	0.1395 (2)	−0.3067 (2)	0.24161 (15)	0.0586 (4)	
C1	1.3707 (4)	0.2513 (3)	0.55842 (19)	0.0833 (8)	
H1A	1.3230	0.1326	0.6002	0.125*	
H1B	1.3738	0.3624	0.6088	0.125*	
H1C	1.5079	0.2718	0.5354	0.125*	
C2	1.2249 (3)	0.2310 (2)	0.44945 (14)	0.0514 (4)	
C3	1.0431 (3)	0.2697 (3)	0.45779 (14)	0.0578 (5)	
H3	1.0116	0.3084	0.5309	0.069*	
C4	0.9072 (3)	0.2521 (2)	0.35961 (14)	0.0468 (4)	
H4	0.7868	0.2807	0.3664	0.056*	
C5	0.95280 (19)	0.19123 (17)	0.25110 (11)	0.0301 (3)	
C6	1.1336 (2)	0.1525 (2)	0.24079 (13)	0.0391 (3)	
H6	1.1648	0.1128	0.1678	0.047*	
C7	1.2684 (3)	0.1733 (3)	0.34053 (15)	0.0508 (4)	
H7	1.3904	0.1478	0.3335	0.061*	
C8	0.58221 (18)	−0.21318 (17)	0.12696 (10)	0.0275 (2)	

### Atomic displacement parameters (Å^2^)

**Table:**

	*U*^11^	*U*^22^	*U*^33^	*U*^12^	*U*^13^	*U*^23^
S1	0.02984 (17)	0.02068 (16)	0.03386 (18)	0.00862 (11)	0.00265 (11)	0.00626 (11)
O1	0.0493 (6)	0.0259 (5)	0.0674 (7)	0.0189 (4)	0.0042 (5)	0.0108 (5)
O2	0.0366 (5)	0.0402 (5)	0.0313 (5)	0.0090 (4)	0.0053 (4)	0.0083 (4)
N1	0.0278 (5)	0.0233 (5)	0.0357 (5)	0.0081 (4)	0.0037 (4)	0.0035 (4)
N2	0.0397 (6)	0.0250 (5)	0.0526 (7)	0.0129 (5)	0.0117 (5)	0.0047 (5)
N3	0.0366 (6)	0.0243 (5)	0.0458 (6)	0.0054 (4)	0.0072 (5)	0.0039 (4)
N4	0.0360 (6)	0.0283 (5)	0.0438 (6)	0.0028 (4)	0.0057 (5)	0.0077 (5)
N5	0.0480 (8)	0.0493 (8)	0.0766 (10)	0.0108 (6)	0.0250 (7)	0.0119 (7)
C1	0.0957 (16)	0.0691 (13)	0.0535 (11)	−0.0045 (12)	−0.0346 (11)	0.0146 (10)
C2	0.0585 (10)	0.0377 (8)	0.0402 (8)	−0.0029 (7)	−0.0128 (7)	0.0082 (6)
C3	0.0735 (12)	0.0533 (10)	0.0311 (7)	0.0038 (8)	0.0057 (7)	−0.0045 (7)
C4	0.0470 (8)	0.0470 (8)	0.0410 (8)	0.0101 (7)	0.0089 (6)	−0.0059 (6)
C5	0.0334 (6)	0.0212 (5)	0.0317 (6)	0.0045 (4)	0.0024 (5)	0.0021 (4)
C6	0.0391 (7)	0.0417 (7)	0.0366 (7)	0.0153 (6)	−0.0015 (5)	−0.0002 (6)
C7	0.0441 (8)	0.0537 (9)	0.0513 (9)	0.0154 (7)	−0.0110 (7)	0.0039 (7)
C8	0.0301 (6)	0.0233 (5)	0.0271 (5)	0.0073 (4)	−0.0009 (4)	0.0036 (4)

### Geometric parameters (Å, °)

**Table:**

S1—O1	1.4324 (10)	C1—H1B	0.9600
S1—O2	1.4387 (10)	C1—H1C	0.9600
S1—N1	1.6064 (10)	C2—C7	1.376 (3)
S1—C5	1.7648 (13)	C2—C3	1.385 (3)
N1—C8	1.3147 (15)	C3—C4	1.384 (3)
N2—C8	1.3104 (17)	C3—H3	0.9300
N2—H2N	0.80 (2)	C4—C5	1.385 (2)
N2—H3N	0.89 (2)	C4—H4	0.9300
N3—N4	1.2523 (17)	C5—C6	1.381 (2)
N3—C8	1.4034 (15)	C6—C7	1.389 (2)
N4—N5	1.112 (2)	C6—H6	0.9300
C1—C2	1.515 (2)	C7—H7	0.9300
C1—H1A	0.9600		
			
O1—S1—O2	117.13 (6)	C3—C2—C1	120.33 (19)
O1—S1—N1	105.65 (6)	C4—C3—C2	121.43 (15)
O2—S1—N1	112.85 (6)	C4—C3—H3	119.3
O1—S1—C5	107.75 (6)	C2—C3—H3	119.3
O2—S1—C5	107.55 (6)	C3—C4—C5	119.15 (16)
N1—S1—C5	105.20 (6)	C3—C4—H4	120.4
C8—N1—S1	121.49 (9)	C5—C4—H4	120.4
C8—N2—H2N	120.4 (15)	C6—C5—C4	120.34 (13)
C8—N2—H3N	119.0 (13)	C6—C5—S1	120.58 (10)
H2N—N2—H3N	120.6 (19)	C4—C5—S1	119.08 (11)
N4—N3—C8	113.81 (11)	C5—C6—C7	119.34 (14)
N5—N4—N3	171.56 (14)	C5—C6—H6	120.3
C2—C1—H1A	109.5	C7—C6—H6	120.3
C2—C1—H1B	109.5	C2—C7—C6	121.31 (16)
H1A—C1—H1B	109.5	C2—C7—H7	119.3
C2—C1—H1C	109.5	C6—C7—H7	119.3
H1A—C1—H1C	109.5	N2—C8—N1	130.52 (12)
H1B—C1—H1C	109.5	N2—C8—N3	111.45 (11)
C7—C2—C3	118.41 (15)	N1—C8—N3	118.03 (11)
C7—C2—C1	121.3 (2)		
			
O1—S1—N1—C8	166.90 (10)	O1—S1—C5—C4	41.58 (13)
O2—S1—N1—C8	37.71 (12)	O2—S1—C5—C4	168.70 (11)
C5—S1—N1—C8	−79.26 (11)	N1—S1—C5—C4	−70.78 (12)
C8—N3—N4—N5	−177.3 (11)	C4—C5—C6—C7	0.6 (2)
C7—C2—C3—C4	−0.2 (3)	S1—C5—C6—C7	−178.38 (12)
C1—C2—C3—C4	−179.94 (16)	C3—C2—C7—C6	−0.5 (3)
C2—C3—C4—C5	1.0 (3)	C1—C2—C7—C6	179.26 (16)
C3—C4—C5—C6	−1.2 (2)	C5—C6—C7—C2	0.3 (2)
C3—C4—C5—S1	177.75 (12)	S1—N1—C8—N2	−2.0 (2)
O1—S1—C5—C6	−139.43 (11)	S1—N1—C8—N3	177.16 (9)
O2—S1—C5—C6	−12.31 (13)	N4—N3—C8—N2	176.19 (12)
N1—S1—C5—C6	108.21 (11)	N4—N3—C8—N1	−3.12 (18)

### Hydrogen-bond geometry (Å, °)

**Table:**

*D*—H···*A*	*D*—H	H···*A*	*D*···*A*	*D*—H···*A*
N2—H2N···O2^i^	0.80 (2)	2.24 (2)	2.9459 (16)	148 (2)
N2—H3N···O1^ii^	0.89 (2)	2.08 (2)	2.9481 (15)	164 (2)
N2—H2N···O2	0.80 (2)	2.34 (2)	2.8862 (16)	127 (2)

Symmetry codes: (i) −*x*+2, −*y*, −*z*; (ii) *x*, *y*−1, *z*.
